# Sensitization of interferon-γ induced apoptosis in human osteosarcoma cells by extracellular S100A4

**DOI:** 10.1186/1471-2407-4-52

**Published:** 2004-08-19

**Authors:** Kjetil Boye Pedersen, Kristin Andersen, Øystein Fodstad, Gunhild Mari Mælandsmo

**Affiliations:** 1 Department of Tumor Biology, The Norwegian Radium Hospital, Montebello, N-0310 Oslo, Norway

## Abstract

**Background:**

S100A4 is a small Ca^2+^-binding protein of the S100 family with metastasis-promoting properties. Recently, secreted S100A4 protein has been shown to possess a number of functions, including induction of angiogenesis, stimulation of cell motility and neurite extension.

**Methods:**

Cell cultures from two human osteosarcoma cell lines, OHS and its anti-S100A4 ribozyme transfected counterpart II-11b, was treated with IFN-γ and recombinant S100A4 in order to study the sensitizing effects of extracellular S100A4 on IFN-γ mediated apoptosis. Induction of apoptosis was demonstrated by DNA fragmentation, cleavage of poly (ADP-ribose) polymerase and Lamin B.

**Results:**

In the present work, we found that the S100A4-expressing human osteosarcoma cell line OHS was more sensitive to IFN-γ-mediated apoptosis than the II-11b cells. S100A4 protein was detected in conditioned medium from OHS cells, but not from II-11b cells, and addition of recombinant S100A4 to the cell medium sensitized II-11b cells to apoptosis induced by IFN-γ. The S100A4/IFN-γ-mediated induction of apoptosis was shown to be independent of caspase activation, but dependent on the formation of reactive oxygen species. Furthermore, addition of extracellular S100A4 was demonstrated to activate nuclear factor-κB (NF-κB).

**Conclusion:**

In conclusion, we have shown that S100A4 sensitizes osteosarcoma cells to IFN-γ-mediated induction of apoptosis. Additionally, extracellular S100A4 activates NF-κB, but whether these events are causally related remains unknown.

## Background

The S100 protein family consists of at least 21 small, acidic, Ca^2+^-binding proteins with different expression patterns and apparently diverse functional and biological properties [1]. The S100A4 protein has been associated with increased metastatic capacity of cancer cells [2-4], but how S100A4 exerts the metastasis-promoting effects is still incompletely understood. S100A4 has been shown to interact with a number of cytoskeleton-associated proteins, including non-muscle myosin [5], actin [6] and non-muscle tropomyosin [7], thereby possibly affecting cell motility. In addition, we have previously demonstrated an association between S100A4, matrix metalloproteinases and the invasive capacity of osteosarcoma cells [8]. It has also been reported that S100A4 can be secreted, and suggested extracellular functions include induction of angiogenesis [9], stimulation of cell motility [10], and stimulation of neurite outgrowth [11]. Moreover, we have recently demonstrated nuclear localization of S100A4, and an association between nuclear expression and tumor stage in colorectal cancer [12], indicating other, yet undiscovered, functions of S100A4. Finally, S100A4 has been reported to interact with the tumor suppressor protein p53 and to modulate transcription of downstream target genes, thus influencing p53-mediated apoptosis [13].

Interferon-gamma (IFN-γ) is a pleiotropic cytokine secreted from activated T lymphocytes and natural killer cells. It can activate and suppress a number of target genes, leading to various effects, including inhibition of viral replication, activation of immune cells and induction of cell cycle arrest and apoptosis [14,15]. Apoptosis is a process of programmed cell death characterized by chromatin condensation and DNA fragmentation, plasma membrane blebbing and cell shrinkage. One of the central executioners of the apoptotic pathway are the caspases [16]. It has, however, become evident that a number of caspase-independent pathways exist, leading to programmed cell death involving cathepsins [17], apoptosis inducing factor [18], calpains, AP24 and other serine proteases [19]. Evidence has been provided showing that IFN-γ is able to induce programmed cell death by both caspase-dependent and -independent pathways, e.g. through cathepsin D [20], caspase-1 [21], TNF-related apoptosis-inducing ligand (TRAIL) [22] and Fas/FasL [23].

Searching for cytokines and signal transduction modulators affecting S100A4 expression, we recently discovered that IFN-γ downregulated S100A4 transcription and induced apoptosis in the human osteosarcoma cell line OHS [24]. In the corresponding anti-S100A4 ribozyme-transfected cell line II-11b [3], apoptosis induction was remarkably lower, and these findings prompted us to investigate whether S100A4 could affect IFN-γ-mediated apoptosis. In the present study, we show that S100A4 can be secreted from these human osteosarcoma cells, and that extracellular addition of rS100A4 sensitizes cells to apoptosis induced by IFN-γ. Furthermore, we demonstrate that this cell death pathway is independent of caspases, but possibly dependent upon NF-κB transactivation and generation of reactive oxygen species (ROS).

## Methods

### Cell culture and treatment

The human osteosarcoma cell line OHS and its anti-S100A4 ribozyme transfected counterpart II-11b have been described previously [8]. Cell lines were cultivated in RPMI-1640 (Bio Whittaker, Verviers, Belgium) containing 10% fetal bovine serum (FBS; Biochrome KG, Berlin, Germany), 1 mM Hepes, and 2 mM Glutamax (GIBCO BRL, Life Technologies, Paisley, UK). For all experiments, subconfluent cultures were trypsinated and seeded at 1.5 × 10^4 ^cells/cm^2^. After overnight incubation, the culture medium was replaced with medium in presence or absence of IFN-γ and recombinant S100 proteins, and harvested as indicated in the text. Where indicated, protease inhibitors or antioxidants were added to the cell culture simultaneously as IFN-γ.

### Materials

Human recombinant IFN-γ, L-NMMA, zVAD-fmk, zYVAD-fmk, zVDVAD-fmk, zDEVD-fmk, zVEID-fmk, zIETD-fmk, zLEHD-fmk, zFA-fmk and E64 were purchased from Calbiochem (San Diego, CA, USA). N-acetylcysteine was from Sigma Chemical Co (St Louis, MO, USA).

### Production of proteins

Histidine-tagged mouse recombinant S100A4 was cloned into the pQE30 vector (kindly provided by E. Lukanidin, Institute of Cancer Biology, Copenhagen, Denmark), expressed in E. coli, purified on a Ni^2+^-column and dialyzed against phosphate-buffered saline. A satisfactory degree of purity was confirmed by SDS polyacrylamide gel electrophoresis (SDS-PAGE) where a single band was visualized by silver staining. Verification of the identity of the protein was performed using matrix-assisted laser desorption ionization-time-of-flight mass spectrometry (MALDI). Human recombinant S100A10 and S100A13 tagged with maltose-binding protein were kindly provided by C. S. Skjerpen (Dep. of Biochemistry, The Norwegian Radium Hospital, Oslo, Norway).

### Cell viability

Cell viability was estimated by measuring metabolic activity using CellTiter 96^® ^AQ_ueous _One Solution Reagent (MTS) (Promega, Madison, WI, USA) according to the manufacturer's manual. The absorbance at 490 nm was recorded using a Wallac 1420 Victor^2 ^Multilabel counter (Wallac Oy, Turku, Finland).

### Western blot analysis

Protein lysates were prepared in 50 mM Tris-HCl (pH 7.5) containing 150 mM NaCl and 0.1% NP-40 with 1 mM PMSF and 2 μg/ml each of pepstatin, aprotinin (Sigma Chemical Co, St Louis, MO, USA) and leupeptin (Roche Diagnostics, Mannheim, Germany). Total protein lysate from each sample was separated on 8–12% SDS-polyacrylamide gel electrophoresis, and transferred onto Immobilon-P membranes (Millipore, Bedford, MA, USA) according to the manufacturer's manual. As a loading and transfer control, the membranes were stained with 0.1% amidoblack. After blockage of non-specific binding sites with 10% non-fat dry milk in Tris-buffered saline with 0.25% Tween (TBST), blots were incubated for 1 h at room temperature with rabbit polyclonal anti-poly (ADP-ribose) polymerase (PARP; diluted 1:1000, Roche Diagnostics, Mannheim, Germany), mouse monoclonal anti-lamin B (0.3 μg/ml; Oncogene Research Products, Boston, MA, USA), mouse monoclonal anti-α-tubulin (0.3 μg/ml; Oncogene Research Products, Boston, MA, USA), rabbit polyclonal anti-caspase-1 (1:100; Santa Cruz Biotechnology, Santa Cruz, CA, USA), goat polyclonal anti-caspase-3 (1:2000; R&D Systems, Minneapolis, MN, USA), mouse monoclonal anti-caspase-8 (1:5000; Alexis Biochemicals, Montreal, Canada), mouse monoclonal anti-caspase-9 (1:1000; R&D Systems, Minneapolis, MN, USA), rabbit polyclonal anti-caspase-12 (1:2000; Oncogene Research Products, San Diego, CA, USA), mouse monoclonal anti-NFκB p65 (1:1000, Cell Signaling Technology Inc. Beverly, MA, USA) or mouse monoclonal anti-phospho-IκBα (Ser32/36) (1:2000, Cell Signaling Technology Inc. Beverly, MA, USA). After washing, the blots were incubated for 1 h at room temperature with a horseradish peroxidase-conjugated secondary antibody (DAKO, Glostrup, Denmark) diluted 1:5000. Anti-lamin B, anti-α-tubulin, anti-PARP, anti-caspase-1, anti-caspase-8, anti-NFκB p65 and anti-phospho-IκBα were diluted in TBST with 5% non-fat dry milk, anti-caspase-3 and anti-caspase-12 were diluted in TBS containing 0.05% Tween and 2% non-fat dry milk and anti-caspase-9 was diluted in TBS with 0.05% Tween, 1% non-fat dry milk and 1% BSA. Signals were visualized using the ECL chemiluminescence substrate (Amersham Pharmacia Biotech, Buckinghamshire, UK).

### DNA fragmentation analysis

Cells were resuspended in lysis buffer (10 mM Tris-HCl pH 7.4, 10 mM NaCl, 10 mM EDTA, 0.5 % SDS and 0.5 μg/ml proteinase K) and incubated for 1 hour at 50°C. High molecular weight DNA was precipitated by adding 1 M NaCl and subsequently incubated overnight at 4°C. Samples were centrifuged for 30 min, 2700 g at 4°C, and supernatants collected. 95 % ethanol was added, and DNA precipitated overnight at -20°C. Samples were centrifuged for 10 min, 10 000 rpm at 4°C, and pellets were washed in 70 % ethanol and re-centrifuged. Thereafter, pellets were air-dried and dissolved in 10 mM Tris-HCl (pH 7.0), 15 mM NaCl, 1 mM EDTA and 0.2 mg/ml RNAse A, and incubated for 1 h at room temperature. DNA was separated by electrophoresis in 1.0 % agarose gel containing ethidium bromide and photographed under ultraviolet illumination.

### Detection of S100A4 in cell culture medium

Cell culture medium from OHS and II-11b cells were harvested at 24, 48 and 72 hours, and immediately filtered using a 0.45 μm filter to discard cells in suspension. From each sample, 1.5 ml of medium was subjected to immunoprecipitation using 15 μg of rabbit polyclonal anti-S100A4 (DAKO, Glostrup, Denmark) or 15 μg rabbit polyclonal anti-p300 (Santa Cruz Biotechnology, Santa Cruz, CA, USA). Primary antibody binding was carried out in cell culture medium at 4°C overnight. Thereafter, antibody was precipitated using Protein A Sepharose beads for 1 h at 4°C and eluted in loading buffer. Samples were subjected to Western blot analysis as described above using rabbit polyclonal anti-S100A4 diluted 1:300 in TBST.

### Transient transfection and plasmid constructs

The NF-κB reporter construct containing three NF-κB response elements (i.e., sites identical to the κB site from the Igκ light chain promoter) driving luciferase expression has been described previously [25]. 1.0 × 10^6 ^II-11b cells were transiently transfected with 10 μg of the NF-κB reporter construct using electroporation (240 V). Thereafter, cells were seeded in 96-well plates at a density of 4.5 × 10^4 ^cells/cm^2 ^and incubated in the absence or presence of rS100A4 and zFA-fmk. Cells were harvested 48 h later and assayed for luciferase activity using the Luciferase Assay System (Promega, Madison, WI, USA) according to the manufacturer's manual.

### Statistical analysis

All statistical analyses were performed using Student's t-test. P-values less than 0.05 were considered to be statistically significant.

## Results

### OHS cells are more sensitive than II-11b cells to IFN-γ-mediated suppression of cell viability

**Figure 1:**
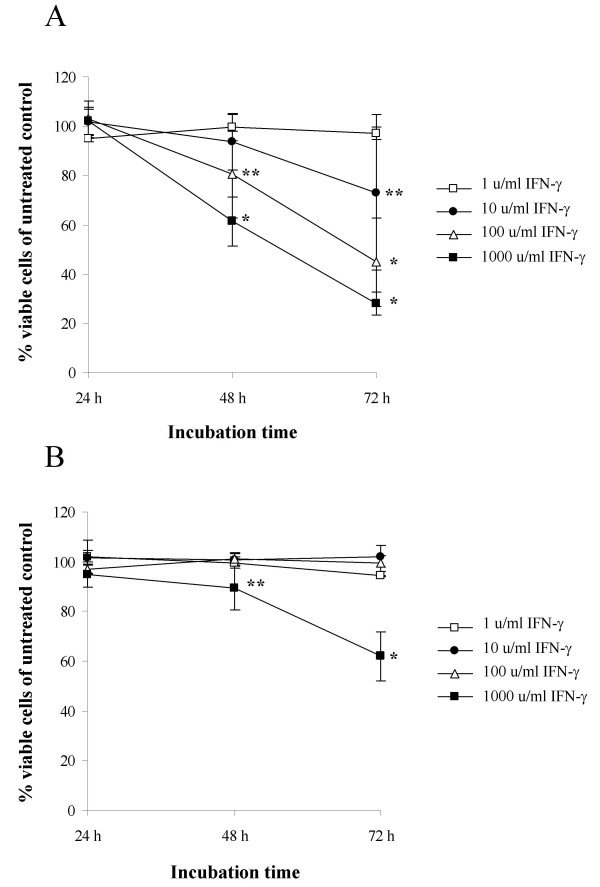
**OHS cells are more sensitive than II-11b cells to IFN-γ-mediated suppression of cell viability. **Cell viability was measured in OHS cells (*A*) and II-11b cells (*B*) after treatment with the indicated concentrations of IFN-γ for 24, 48 and 72 hours. In each experiment, untreated control cells were included at all time points, and all values are given as a percentage of corresponding untreated control cells. The results are presented as mean values ± S.D. of at least three independent experiments performed in triplicate. *, p < 0.0001 and **, p < 0.05 as compared to untreated control cells.

We have previously observed that the human osteosarcoma cell line OHS is more sensitive to IFN-γ induced apoptosis than the S100A4-ribozyme transfected counterpart II-11b [24]. To examine the effects of IFN-γ on cell viability in more detail, time- and dose-response experiments were performed (Fig. 1A and 1B). In OHS cells, addition of 1 u/ml IFN-γ had no effect on cell viability, while an increasing amount of cell death was observed with higher concentrations. On the other hand, II-11b cells only showed a marked reduction in cell number after treatment with 1000 u/ml IFN-γ for 72 hours.

### IFN-γ induces apoptosis in OHS Cells

**Figure 2:**
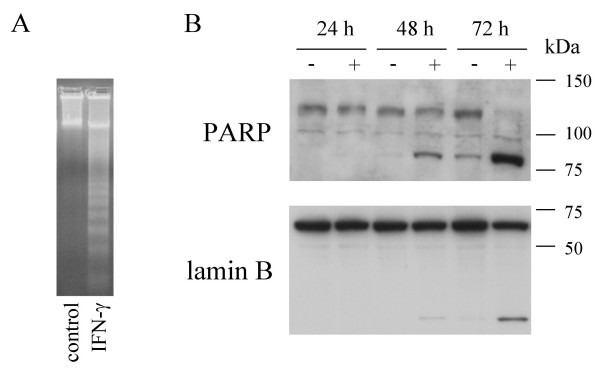
**DNA fragmentation, PARP and Lamin B cleavage in IFN-γ-treated OHS cells. ***A*, Ethidium bromide stained agarose gel of DNA isolated from OHS cells grown with or without 100 u/ml IFN-γ for 72 hours. *B*, Western blot analysis of the expression of PARP and Lamin B in total cell lysates. OHS cells were left untreated (-) or treated with 100 u/ml IFN-γ (+) for 24, 48 and 72 hours, and lysates subjected to Western blot analysis as described in the "Materials and Methods" section.

Previously, we have shown that treatment of OHS cells with IFN-γ induced a significant increase in the fraction of apoptotic cells, while the cell cycle distribution remained unchanged [24]. Thus, it seemed plausible that the reduced cell viability could be due to induction of apoptosis, and to confirm this hypothesis, we investigated some of the known hallmarks of the apoptotic process. As seen in Fig. 2A, IFN-γ induced DNA fragmentation in OHS cells. Additionally, treatment with IFN-γ induced cleavage of poly (ADP-ribose) polymerase (PARP) and lamin B (Fig. 2B). Taken together, these results clearly demonstrated that IFN-γ-treatment induces apoptosis in OHS cells.

### Detection of S100A4 in cell culture medium

**Figure 3:**
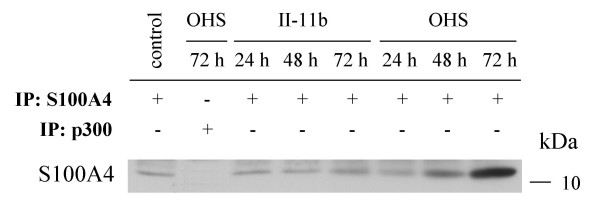
**OHS cells secrete S100A4. **Immunoprecipitation of cell culture medium alone (control) or cell culture medium from II-11b and OHS cells cultured for 24, 48 and 72 hours as indicated. 1.5 ml of medium was immunoprecipitated using rabbit polyclonal anti-S100A4 or anti-p300, and subsequently immunoblotted with anti-S100A4.

When performing the OHS cell culture experiments, we observed that, when keeping the concentration of IFN-γ constant, decreasing the volume of cell culture medium or increasing the number of cells resulted in increased cell death (data not shown). This observation suggested that the cells might secrete a soluble factor with impact on the level of apoptosis induced by IFN-γ. Since the II-11b cells are derived from the OHS cells by transfection with an S100A4-specific ribozyme, this finding prompted us to investigate whether S100A4 is secreted from these cells, and whether extracellular S100A4 could cooperate with IFN-γ in induction of apoptosis. Fig. 3 shows an immunoprecipitation of cell culture medium from OHS and II-11b cells grown for 24, 48 and 72 hours. This clearly demonstrated that S100A4 is present in cell culture medium from OHS cells, and to a much lesser extent from II-11b cells. A small amount of S100A4 was also detected in the cell culture medium control. This possibly reflects bovine S100A4 present in fetal calf serum added to the medium, but the exact nature of this signal has not been further investigated. To exclude cell lysis as the cause of extracellular S100A4, lactate dehydrogenase activity was measured in the conditioned culture medium. No significant increase in enzyme activity was detected, supporting the hypothesis that S100A4 is actively secreted (data not shown). Importantly, cell culture medium from OHS cells cultured for 72 hours immunoprecipitated with a rabbit polyclonal anti-p300 antibody did not give any S100A4-signal, indicating that the detection of S100A4 was not due to unspecific binding.

### Sensitization of IFN-γ-mediated apoptosis by extracellular S100A4

**Figure 4:**
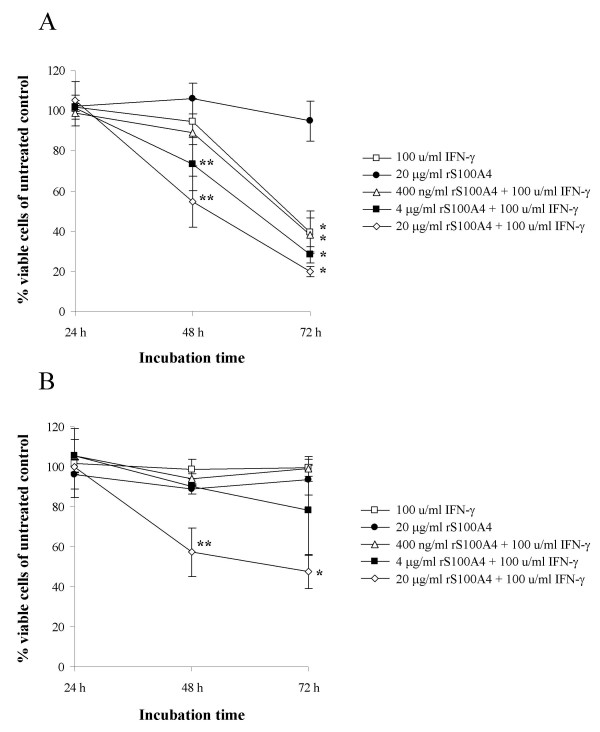
**Addition of recombinant S100A4 sensitizes OHS and II-11b cells to IFN-γ-induced apoptosis. **Cell viability was measured in OHS and II-11b cells after addition of the indicated concentrations of recombinant S100A4 to the cell culture medium with or without 100 u/ml IFN-γ. All values are given as a percentage of viable cells relative to untreated control cells. The results are presented as mean values ± S.D. of at least three independent experiments performed in triplicate. *, p < 0.0001 and **, p < 0.05 as compared to untreated control cells.

**Figure 5:**
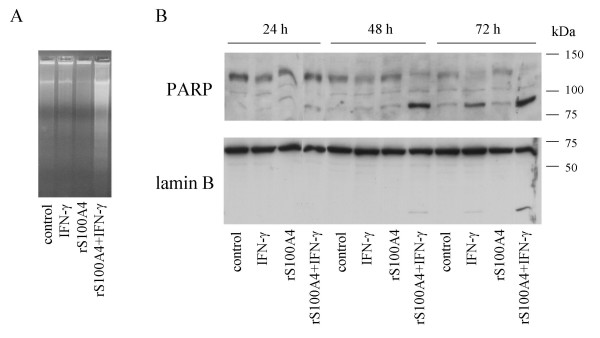
**DNA fragmentation, PARP and Lamin B cleavage in rS100A4/IFN-γ-treated II-11b cells. ***A*, Ethidium bromide stained agarose gel of DNA isolated from II-11b cells. Cells were stimulated or left untreated with 100 u/ml IFN-γ for 72 hours in the presence or absence of 20 μg/ml rS100A4 as indicated. *B*, Western blot analysis of the expression of PARP and Lamin B in total cell lysates. II-11b cells were left untreated or treated with 100 u/ml IFN-γ and/or 20 μg/ml rS100A4 for 24, 48 and 72 hours as indicated, and lysates subjected to Western blot analysis as described in "Materials and Methods".

In order to investigate whether extracellular S100A4 could increase apoptosis induced by IFN-γ, recombinant S100A4 (rS100A4) was added to the cell culture medium of OHS and II-11b cells in addition to IFN-γ. Addition of rS100A4 alone had no effect on cell viability at concentrations ranging from 400 pg/ml to 20 μg/ml (Fig. 4 and data not shown). On the other hand, when 20 μg/ml of rS100A4 was added together with 100 u/ml IFN-γ to II-11b cells, a significant decrease in cell viability was observed (Fig. 4B). In fact, when II-11b cells were incubated with 20 μg/ml rS100A4, the level of apoptosis induced by IFN-γ was similar as in OHS cells. Addition of rS100A4 to OHS cells also increased the IFN-γ-mediated cell death, thus seemingly adding to the effects of the endogenously produced extracellular S100A4 (Fig 4A). Accordingly, a significant decrease in cell viability was seen at lower concentrations of added rS100A4 in OHS cells than in II-11b cells. Furthermore, we made attempts to neutralize the secreted S100A4 protein by adding anti-S100A4 antibodies to the cell culture medium of IFN-γ treated OHS cells, but were unable to detect any decrease in the amount of cell death (data not shown). Possibly, the antibodies used (DAKO, Glostup, Denmark, as well as three in-house produced monoclonal antibodies [26]) were not able to block the activity of extracellular S100A4. S100 proteins share a high degree of sequence homology; therefore it was of interest to investigate whether other S100 proteins were able to sensitize tumor cells to IFN-γ-mediated cell death. Addition of 20 μg/ml recombinant S100A10 or S100A13 had no effect on apoptosis induction by IFN-γ in II-11b cells. Thus, it seems unlikely that the observed effects are a general feature of S100 proteins, but whether other S100 proteins than those tested here could possess IFN-γ-sensitizing properties requires further investigation. The possibility exists that rS100A4 and IFN-γ induce a different type of cell death in II-11b cells than the IFN-γ-mediated apoptosis in OHS cells. DNA fragmentation (Fig. 5A) as well as PARP and Lamin B cleavage (Fig. 5A) were demonstrated also in II-11b cells, suggesting that addition of recombinant S100A4 actually mimics the biological effects of endogenous S100A4 in these experiments.

### IFN-γ-mediated apoptosis in OHS dells is caspase-independent

**Figure 6:**
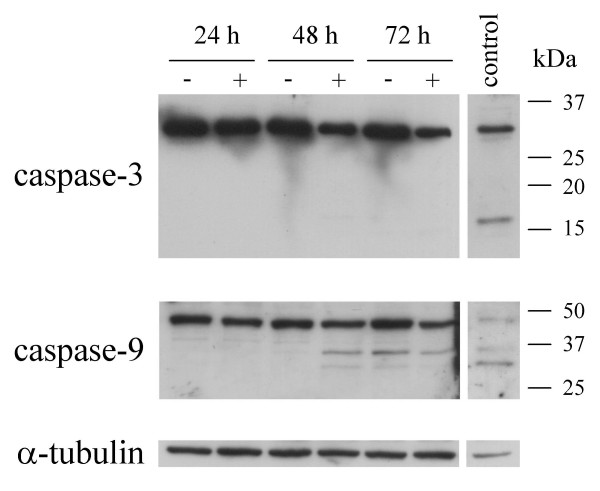
**Western blot analysis of caspase-3 and caspase-9 in total cell lysates. **OHS cells were left untreated (-) or treated with 100 u/ml IFN-γ (+) for 24, 48 and 72 hours, and lysates subjected to Western blot analysis as described in "Materials and Methods". As a positive control for caspase activation, an immunotoxin-treated breast cancer cell line was included. α-tubulin serves as a loading control. The results shown are representative of three independent experiments.

**Figure 7:**
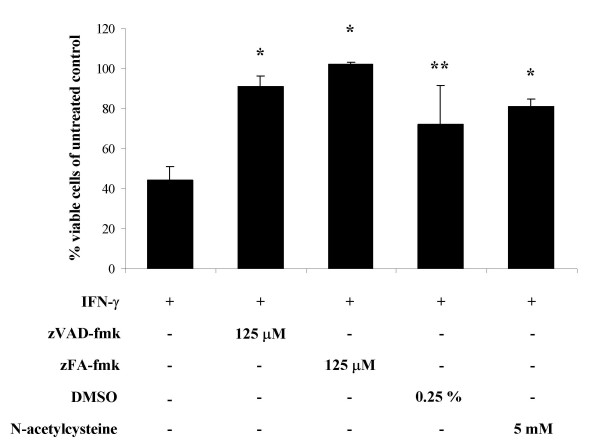
**Inhibition of cell death by zVAD-fmk, zFA-fmk, DMSO and NAC. **OHS cells were cultivated in the presence or absence of 100 u/ml IFN-γ and the indicated inhibitors for 72 hours, and cell viability was measured as described in "Materials and Methods". All values are given as a percentage of viable cells relative to corresponding cells treated with the indicated inhibitor, but without IFN-γ-treatment. Data are mean values ± S.D. The results represent at least three independent experiments performed in triplicate. *, p < 0.001 and **, p < 0.05 as compared to IFN-γ-treated cells without inhibitor.

Caspase-mediated apoptosis is the most important program of cell death. In order to investigate whether IFN-γ activated the caspase cascade in our cell system, we have followed two different approaches: detection of activated caspases by Western blotting and treatment of cell cultures with caspase inhibitors. Using specific antibodies detecting both the proenzyme and the active form of caspase-1, -3, -8, -9 and -12, we were not able to detect any activation of caspase-1, -3, -8 or -12 (Fig. 6 and data not shown). However, the caspase-9 antibody detected a weak, but distinct, ~37 kDa band in IFN-γ treated cells at 48 and 72 h and also in control cells at 72 h. This probably corresponds to one of the active forms of caspase-9. Additionally, we observed a lower level of expression of the proenzyme of caspase-3 in IFN-γ treated cells at 48 and 72 h (Fig. 6). This could possibly indicate a suppression of caspase-3 expression by IFN-γ or activation of caspase-3 with resulting cleavage of the proenzyme. On the other hand, no active forms of caspase-3 were detected, even when exposing the film for several hours. To ensure that the antibodies were able to detect the mature forms, immunotoxin-treated breast cancer cells were included as a positive control [27]. Furthermore, the pan-caspase inhibitor zVAD-fmk inhibited IFN-γ-induced cell death, albeit only at high concentrations (Fig. 7). zVAD-fmk suppressed IFN-γ-mediated apoptosis significantly at 125 μM, whereas 50 μM inhibited cell death non-significantly, and 10 μM zVAD-fmk had no effect on apoptosis inhibition. The latter concentration has been reported to be sufficient to inhibit most caspases [28], therefore it seems likely that the observed effect of zVAD-fmk could be due to inhibition of other proteases or signaling events than caspases. In addition, inhibition of caspase-1 (zYVAD-fmk), caspase-2 (zVDVAD-fmk), caspase-3 and -7 (zDEVD-fmk), caspase-6 (zVEID-fmk), caspase-8 (zIETD-fmk) and caspase-9 (zLEHD-fmk) had no effect on IFN-γ-induced cell death at concentrations up to 50 μM. Taken together, we have not observed any activation of caspases-1, -3, -8 or -12, whereas a low-level activation of caspase-9 was detected. Inhibitors against a range of caspases including caspase-9 did not suppress induction of cell death; hence, from these experiments we concluded that the cell death program induced by IFN-γ in OHS cells most likely is caspase-independent.

### Reactive oxygen species mediate IFN-γ-induced apoptosis

The caspase inhibitors used in the experiments above were solved in dimethyl sulfoxide (DMSO), and therefore DMSO was included as a negative control. Surprisingly, a significant inhibition of IFN-γ-induced apoptosis was observed using 0.25 % DMSO (Fig. 7). One of the known biological properties of DMSO is scavenging of hydroxyl radicals, indicating that the generation of reactive oxygen species (ROS) could be implicated in the observed induction of apoptosis. Furthermore, treatment with the commonly used antioxidant N-acetylcysteine significantly suppressed the IFN-γ-induced cell death (Fig. 7), whereas the nitric oxide synthase inhibitor L-NMMA had no effect (data not shown). Notably, zVAD-fmk was recently shown to possess potent antioxidant effects at high concentrations [29] possibly explaining its inhibitory effects shown above.

### IFN-γ-mediated apoptosis in OHS cells is independent of cathepsin B, but possibly mediated by NF-κB

**Figure 8:**
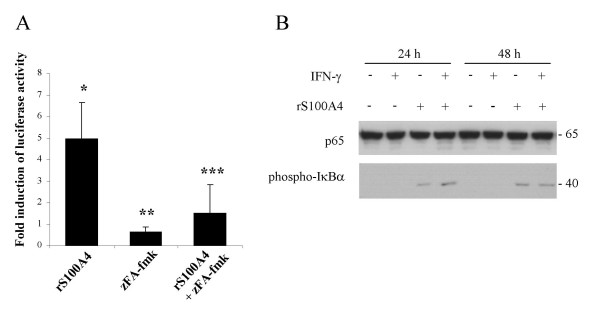
**Recombinant S100A4 induces NF-κB transactivation. ***A*, II-11b cells were transiently transfected with an NF-κB reporter construct and incubated with or without 4 μg/ml rS100A4 and 50 μM zFA-fmk for 48 hours. Luciferase activity was expressed as fold induction of activity compared to the corresponding untreated control. Data are mean values ± S.D. The results represent at least three independent experiments performed in duplicate. *, p < 0.001 and **, p < 0.01 as compared to untreated control. ***, p < 0.005 as compared to rS100A4-treatment without zFA-fmk. *B*, Western blot analysis of NF-κB p65 and phospho-IκBα (Ser32/36) in total cell lysates from II-11b cells, untreated or treated with 100 u/ml IFN-γ and/or 20 μg/ml rS100A4 for 24 and 48 hours as indicated.

The pan-caspase inhibitor zVAD-fmk has previously been shown to bind the cysteine protease cathepsin B and to suppress cathepsin B activity *in vitro *[30]. Since cathepsins are able to act as mediators of programmed cell death [17], zVAD-fmk could possibly inhibit IFN-γ-induced apoptosis through inhibition of cathepsin B. However, 50–250 μM E64 (a cysteine protease inhibitor) or 1–10 μM zFA-fmk (a cathepsin B inhibitor) failed to suppress induction of the observed cell death (data not shown), arguing against cathepsin B as a mediator of IFN-γ-induced apoptosis. On the other hand, high concentrations (125 μM) of zFA-fmk completely inhibited induction of apoptosis (Fig. 7). Cathepsin B activity is most likely suppressed at concentrations below 10 μM [28]; thus, we believe that the observed inhibition by zFA-fmk is due to non-specific effects. Indeed, zFA-fmk was recently shown to suppress transactivation by NF-κB at concentrations similar to those used in these experiments [30]. Interestingly, addition of rS100A4 to II-11b cells lead to a marked induction of NF-κB activity in transient transfection assays using an NF-κB reporter construct, and this induction was blocked by zFA-fmk (Fig. 8A). Furthermore, IκBα was phosphorylated upon addition of extracellular S100A4 (Fig. 8B), confirming activation of the NF-κB pathway. Taken together, these results demonstrate that extracellular S100A4 activates NF-κB, and suggest that the apoptosis induced by S100A4 and IFN-γ could be mediated through an NF-κB-dependent pathway, but whether these two events are actually causally connected remains to be investigated.

## Discussion

IFN-γ induces apoptosis in a variety of different cell types, but some cells are less susceptible or even resistant to IFN-γ-mediated apoptosis. The reasons for this variation have not been clearly elucidated, and given the biological importance of IFN-γ, a better understanding of the mechanisms of IFN-γ-induced cell death is warranted. In the present study, we have demonstrated that the Ca^2+^-binding protein S100A4 sensitizes human osteosarcoma cells to IFN-γ-induced apoptosis. Furthermore, we have shown that the IFN-γ-mediated cell death is independent of caspases, but possibly mediated by reactive oxygen species. Finally, we have demonstrated that extracellular S100A4 can activate NF-κB through increased phosphorylation of IκBα, but whether this NF-κB transactivation is required for apoptosis induction remains unknown.

Several of the S100 proteins are released into the extracellular space, mostly by unknown mechanisms [1]. S100A4 secretion by human osteosarcoma cells is in agreement with previous reports showing that S100A4 can be released from non-malignant as well as tumor cells [9,31,32]. The apoptosis-inducing properties of extracellular S100A4 are, however, novel findings, emphasizing the multiple functions of this protein. Other extracellular S100 proteins also exert multiple effects, including proinflammatory activity, chemotaxis, neurite extension, stimulation of angiogenesis, and regulation of cell survival [1]. Proapoptotic activity has, to our knowledge, only been demonstrated previously for S100A1, S100B [33] and S100A8/S100A9 [34]. S100B induces apoptosis by interaction with the multi-ligand cell surface receptor RAGE (a member of the immunoglobulin superfamily) [33]. RAGE also binds S100A12 [35], whereas no cell surface receptor for S100A4 has been identified so far. In view of the significant homology between S100 proteins, S100A4 could possibly bind to and activate RAGE. However, treatment with anti-RAGE IgG [35] up to 50 μg/ml had no inhibitory effect on apoptosis induction (data not shown), and higher concentrations were toxic to the cells. Hence, another cell surface receptor might mediate S100A4-induced sensitization of apoptosis, and in line with this, RAGE-independent actions were recently demonstrated for S100B [36]. Notably, mechanisms distinct from ligand-receptor interactions can also propagate proapoptotic signals. S100A8/S100A9 induced apoptosis even when a dialysis membrane hindered contact with the cells, possibly by binding Zn^2+ ^or other divalent cations [34].

Addition of 20 μg/ml rS100A4 (1.5 μM) to the cell medium of II-11b cells was required to induce apoptosis upon IFN-γ-treatment. Comparably, micromolar amounts of S100B and S100A8/A9 were needed to induce cell death [33,34]. Also S100A4-mediated neurite extension [11] as well as stimulation of angiogenesis [9] were shown to require micromolar concentrations of recombinant protein. Whether native extracellular S100A4 is present at these concentrations in our experiments is unknown, as secreted S100A4 was not quantitatively estimated.

In many cases, stimulation of tumor cells with a single proapoptotic signal is not sufficient for induction of programmed cell death. On the other hand, a number of cytokines and other proapoptotic signal substances can increase the cells' sensitivity to other apoptosis-inducing agents, thus initiating the cell death program. IFN-γ induces apoptosis in synergism with a wide variety of such agents, and particularly members of the tumor necrosis factor (TNF) superfamily have been studied, e.g., TNF-α [37], FasL [38], LIGHT [39] and TRAIL [22]. Our work adds a novel proapoptotic signaling molecule to the list of agents acting in synergism with IFN-γ to induce programmed cell death. The mechanisms by which S100A4 sensitizes the tumor cells to apoptosis induction are not completely elucidated. Recently, Grigorian *et al *demonstrated that S100A4 interacts with the tumor suppressor protein p53 to enhance p53-dependent apoptosis. The proposed mechanism was based on modulation of p53-dependent transactivation of target genes [13]. The osteosarcoma cells used in our experiments harbor, however, a mutated p53 protein [40], thus, S100A4 seemingly enhance apoptosis through at least two distinct mechanisms. One possible explanation for the apoptosis sensitizing effects of S100A4 is that the protein positively modulates IFN-γ-signaling in general. By studying the regulation of S100A4 expression, we have demonstrated that extracellular S100A4 enhances IFN-γ-mediated suppression of S100A4 transcription (Pedersen *et al.*, in preparation). These findings, in addition to the present work, suggest that secreted S100A4 could sensitize IFN-γ-signaling, and this possibility is currently under investigation. Previously identified sensitizers of IFN-γ-signaling include type I interferons (IFN-α/-β) [41] and IFN-γ itself [42]. Hu *et al*. discusses, based on their experiments using peripheral blood mononuclear cells, that other factors probably also exist to sensitize IFN-γ-signaling. Interestingly, S100A4 is strongly expressed by T lymphocytes , but whether lymphocytes also secrete S100A4 remains to be investigated.

Our results suggest that S100A4-mediated NF-κB activation might be required for triggering the apoptotic process induced by IFN-γ. To support this hypothesis, we made attempts to inhibit NF-κB signaling by treatment with SN50, a peptide that contains the p50 NLS and selectively blocks nuclear translocation of NF-κB, but this lead to an increased fraction of apoptotic cells. Schotte *et al. *suggest that zFA-fmk has a promoter-specific NF-κB inhibitory effect [30], whereas SN50 potently blocks all NF-κB activity, possibly explaining the divergent results. In general, activation of NF-κB is regarded as an anti-apoptotic event and as such, a total suppression of NF-κB activity could be deleterious to cells. However, NF-κB apparently has a dual role in apoptosis regulation, and cell death induction by NF-κB has been reported in a variety of cell systems [43].

IFN-γ-induced apoptosis in OHS cells was shown to be caspase-independent by two different approaches: (i) no active forms of a number of caspases were detected; and (ii) caspase inhibitors were not able to suppress cell death triggered by IFN-γ. Even though the caspase cascade was not activated, well-known hallmarks of apoptosis were identified. DNA fragmentation and Lamin B cleavage have previously been shown to occur independent of caspase activation [44,45], whereas PARP cleavage has been regarded as a caspase-indispensable event. Cleavage of PARP to an 89-kDa fragment was however evident, but the observed low level activation of caspase-9 was, due to the lack of effect when adding the specific caspase-9 inhibitor, not considered crucial, possibly implicating other proteases than caspases in PARP inactivation. Finally, experiments using the antioxidant NAC indicated that ROS are involved in S100A4/IFN-γ-mediated triggering of apoptosis. In light of recent reports demonstrating that both S100B and S100A8/S100A9 induced apoptosis in an oxidant-dependent manner this result is highly interesting [33,46].

## Conclusions

In conclusion, we have demonstrated that extracellular S100A4 induces NF-κB transactivation and possesses proapoptotic activity through sensitization of IFN-γ-induced apoptosis, but whether activation of NF-κB is required for the observed sensitization of cell death is still unknown. Even though the precise physiological role of the apoptosis sensitizing effect of S100A4 awaits further studies, it highlights yet another area of research in the attempts to elucidate the complex biological roles of S100A4.

## Competing interests

None declared.

## Authors' contributions

KBP carried out the cell culture experiments, immunoprecipitations and western blots used in the detection of S100A4 in cell culture medium. In addition he did the dose-response experiments in II-11b cells, all the experiments using caspase inhibitors and NAC, western blot analysis of caspase activity, and drafted the manuscript. KA carried out dose-response experiments in OHS cells, and in addition all the cell culture experiments used in subsequent western blot analysis of caspase- and NF-κB activity and DNA fragmentation analysis. In addition she did transient transfections and all cell culture experiments using other S100 proteins and anti-RAGE antibodies. ØF participated in the design of the study. GMM conceived the study, and participated in its design and coordination. All authors read and approved the final manuscript.

## Pre-publication history

The pre-publication history for this paper can be accessed here:
